# Limited Short-Term Effects of Tactile Stimulation on the Welfare of Newborn Nellore Calves

**DOI:** 10.3390/vetsci12040393

**Published:** 2025-04-21

**Authors:** Mariana Parra Cerezo, Victor Brusin, Pedro Henrique Esteves Trindade, Adalinda Hernández, Jens Jung, Charlotte Berg, Mateus José Rodrigues Paranhos da Costa

**Affiliations:** 1 Programa de Pós-Graduação em Ciência Animal, Faculdade de Ciências Agrárias e Veterinárias, UNESP, Jaboticabal 14884-900, Brazil; marianaparra9811@gmail.com; 2 Grupo de Estudos e Pesquisas em Etologia e Ecologia Animal (ETCO), Faculdade de Ciências Agrárias e Veterinárias, UNESP, Jaboticabal 14884-900, Brazil; victorhenrique.bezer@ucalgary.ca; 3 Faculty of Veterinary Medicine, University of Calgary, Calgary, AB T2N 1N4, Canada; 4 Department of Large Animal Clinical Sciences, Michigan State University, East Lansing, MI 48824, USA; pesteve@ncsu.edu; 5 Department of Animal Environment and Health, Swedish University of Agricultural Sciences, 532 23 Skara, Sweden; adalinda.hernandez@slu.se (A.H.); jens.jung@slu.se (J.J.); lotta.berg@slu.se (C.B.); 6 Departamento de Zootecnia, Faculdade de Ciências Agrárias e Veterinárias, UNESP, Jaboticabal 14884-900, Brazil; 7 CNPq, Conselho Nacional de Desenvolvimento Científico e Tecnológico, Brasília 71605-001, Brazil

**Keywords:** cow–calf operation, human–animal interaction, heart rate, average daily gain, qualitative behavior assessment

## Abstract

This study aimed to evaluate the effects of tactile stimulation on calf welfare. A total of 54 Nellore calves were assessed, with 28 of them receiving tactile stimulation (WTS) for ~60 s and 26 serving as a control. Five body movements and seven facial expressions were scored. Heart rates (HRs) were recorded in three situations: when the calves were placed in lateral recumbency (HR1), during identification procedures (HR2), and after completion of identification procedures (HR3). The differences between HR3 and HR1, as well as HR3 and HR2 were calculated. Initial and weaning weights were recorded, and ADG and weaning weights adjusted to 240 days were determined. Tactile stimulation significantly influenced “head movements”, “third eyelid” exposure, “eye-opening”, and “strained nostrils”. Except for “strained nostrils”, WTS calves exhibited higher scores in these behavioral categories. Treatment also influenced the difference between HR3 and HR2 (*p* < 0.05) and showed a trend for HR3 and the difference between HR3 and HR1 (*p* < 0.06). A qualitative behavior assessment (QBA) was applied using facial expressions. Two main principal components were identified, PC1 explaining 63.01% of the data variance and reflecting the calves’ emotionality, and PC2 explaining 19.88% and reflecting excitability. Most WTS calves displayed positive emotional states and high excitability, whereas most NTS calves exhibited the opposite. Treatment did not significantly impact PC1 and PC2 indexes and long-term performance indicators (*p* > 0.05). We conclude that tactile stimulation of newborn Nellore calves during their initial handling has the potential to enhance their short-term welfare, but only to a limited extent.

## Simple Summary

Previous results indicate that tactile stimulation enhances animal welfare and human well-being when applied at a young age. This study aimed to evaluate the effects of tactile stimulation on the welfare of Nellore calves. A total of 54 Nellore calves were observed, 28 of which received tactile stimulation (WTS) for ~60 s, while 26 did not (NTS). The calves’ behavior was assessed through body movements and facial expressions scoring. Heart rates (HRs) were recorded in three situations: when the calves were placed in lateral recumbency (HR1), during identification procedures (HR2), and after completion of identification procedures (HR3). Average daily gain and weaning weight adjusted to 240 days were calculated. Behavioral analyses revealed that most WTS calves exhibited positive emotional states and high excitability, whereas NTS calves displayed the opposite. WTS calves also had lower HR3 values and higher absolute difference between HR3 and HR1 (*p* < 0.06), as well as between HR3 and HR2 (*p* < 0.05) compared to NTS calves. However, long-term performance indicators did not differ between WTS and NTS calves (*p* > 0.05). We conclude that tactile stimulation during the initial handling of newborn Nellore calves likely promotes their short-term welfare, but only to a limited extent.

## 1. Introduction

Tactile stimulation during the early stages of life in both animals and humans has been identified as a potential practice to promote animal welfare and human well-being. This is supported by studies conducted on dairy calves [1,2], piglets [3], foals [4], mice [5], and human babies [6]. It is recognized as an important mediator of oxytocin release [7,8], leading to positive effects on the development, behavior, and health in both animals and humans [9,10]. Additionally, tactile stimulation enhances interactions with domestic animals, reducing fear and stress, which facilitates the handling of farm animals [11,12,13].

Tactile stimulation was defined by Mychasiuk et al. [14] as “a positive initial experience that mimics maternal licking and grooming”. In this context, it involves systematic massage with moderate pressure, typically applied to areas of the body where cows naturally stimulate their calves, such as the ventral neck, withers, and head, with less emphasis on the posterior and dorsal regions [15,16]. This physical contact generates stimuli that activate various skin receptors, creating a sensation of comfort. These stimuli are transmitted through somatic afferent pathways to the central nervous system, where they support neural development, with the formation of new synapses [14,17]. Such permanent changes in the brain [18] could explain the increase in growth rate in animals and humans [19].

There is growing interest in understanding and implementing practices that enhance animal welfare by promoting positive experiences, ensuring greater comfort, and fostering positive emotional states [20]. In general, neonatal farm animals become conscious at or shortly after birth, as dramatically increased blood oxygen levels trigger such functions [21]. As a result, depending on the intensity, duration, or frequency of external stimuli, experiences can be perceived as harmful and aversive or positive and rewarding from the earliest days of life. In this regard, tactile stimulation, when used as a form of gentle handling, may mimic the maternal licking that occurs during the first hours of life—characterized as allogrooming or social grooming—which increases oxytocin levels and provides a positive experience for the animal [22,23].

In dairy calf management, tactile stimulation is relatively easy to implement and is considered a beneficial practice. For example, Silva-Antunes and Paranhos da Costa [1] reported positive outcomes from good handling practices, including brushing dairy calves for five minutes during morning milk feeding. This practice was associated with the reduction in antibiotic treatments and calf mortality. The authors attributed these improvements primarily to proper colostrum intake and better housing conditions. They also emphasized that tactile stimulation enhanced the interaction between cowhands and calves, allowing for early detection of clinical signs of diseases and facilitating timely veterinary intervention, when necessary.

However, in beef-producing cow–calf operations, opportunities for routine tactile stimulation are limited, as calves remain with their mothers most of the time and have minimal human contact. The primary difference in the application of tactile stimulation between dairy and beef calves lies in the opportunity for human contact rather than in breed differences. Additionally, most calf routine handling procedures—such as vaccination, medication, castration, and disbudding—are aversive [24,25,26]. Nevertheless, the results of De Passillé et al. [27] showed that adult cows can discriminate between people who treated them aversively from those who handle them gently. This principle could certainly be applied to cow–calf operations, assuming that the quality of human–cattle interactions is critical to the calves’ subsequent response to humans and that aversive handling methods should be avoided.

To our knowledge, few studies have specifically examined the effects of positive tactile stimulation on the welfare of beef calves. Therefore, this study aimed to evaluate the impact of tactile stimulation combined with good handling practices on the welfare of newborn Nellore beef calves. We hypothesized that even when applied infrequently and for short duration, tactile stimulation would have a positive impact on the welfare of beef calves.

## 2. Materials and Methods

### 2.1. Animals and Handling Procedures

This study was conducted at the Agropecuária Orvalho das Flores farm, located in the municipality of Araguaiana, in the state of Mato Grosso, Brazil. Fifty-four Nellore calves were evaluated, all born between August and September 2019.

Newborn calves’ handling procedures followed best practices, as described by Paranhos da Costa et al. [28]. The calves remained on pastures with their dams until weaning, with free access to water and a mineral supplement for calves (Fosbovinho, Tortuga^®^, DSM, Campinas, SP, Brazil). Calves were randomly assigned to one of two treatments: WTS (with tactile stimulation), where calves received tactile stimulation during the handling procedures (n = 28, 11 males and 17 females), and NTS (no tactile stimulation—control group), where calves did not receive tactile stimulation (n = 26, 9 males and 17 females).

All calves in this study were from single and eutocic deliveries and exhibited high vigor (being able to promptly follow their mothers when guided by the cowhands from the maternity pasture to the maternity handling area or corral).

### 2.2. Handling Procedures for Pregnant Cows and Newborn Calves

The farm follows a well-established management protocol for pregnant cows and newborn calves. In the final third of gestation, cows are moved to the maternity pastures, where they are monitored twice daily (early morning and late afternoon) to track deliveries and assist cows experiencing calving difficulties.

Each morning during the routine monitoring, the cowhands carefully observed the cow–calf dyads. Handling was limited to dry calves that either stood up when the cowhands approached or when they were already standing near their mothers. Calves that were still wet or unable to stand up were left undisturbed.

For calves that were already standing and able to follow their mothers, handling began with a cowhand, on horseback, looping a rope around the calf’s neck and holding it. Another cowhand, on foot, then manually restrained the calf, removed the rope, fitted an elastic collar (linked to the mother’s identification number), and performed the first navel disinfection using a commercial solution (Umbicura^®^, Pecuarista D’Oeste Saúde Animal Ltd., Araçatuba, SP, Brazil). During these procedures, the cowhand on horseback positioned himself between the mother and the calf handler to minimize the risk of accidents.

At the end of these procedures, the calf was released, and the cow–calf pair remained in the maternity paddock for three to five days. The handling of calves born in the morning was performed in the afternoon of the same day, while calves born in the afternoon or evening were handled the following morning.

Three and five days after birth, cow–calf dyads were herded to the maternity handling area or corral, depending on their proximity to the maternity pasture. Herding was performed on horseback, ensuring a calm approach without forcing the cows and calves forward. In the handling area or corral, cows were separated from their calves and moved to an adjacent pen, maintaining visual, olfactory, and auditory contact. This separation improves cowhand safety by reducing the risk of accidents due to maternal protective behavior [29].

After the separation, one cowhand approached a calf, restrained it by hand, and either carried it or directed it to the area where handling procedures were performed. There, each calf was placed in lateral recumbency on a cushion, and the cowhand restrained it without applying excessive pressure. The cowhand then removed the elastic collar and applied tactile stimulation to WTS calves (for approx. 30 s), while another handler recorded the calf’s identity and prepared for identification procedures, navel dipping, and subcutaneous antiparasitic injection. Tactile stimulation was repeated (for another approx. 30 s) after identification procedures. The exact duration of tactile stimulation was defined by the opportunity the cowhand holding the calf had to perform it.

Tattoo application was performed with four-position tattoo pliers (Stone Manufacturing and Supply Company, Kansas City, MO, USA). Green tattoo paste (Ketchum^®^, Ketchum Mfg. Co., Inc., Lake Luzerne, NY, USA) was applied inside the right ear (above the upper cartilage ridge) to tattoo the calf’s identification number, and the same process was repeated on the left ear for the mother’s identification number. The tattooed areas were then covered with tattoo paste for better visibility.

After tattooing, the cowhand punched two small holes (Allflex Awl, 6 mm, Allflex Livestock Intelligence, Joinville, SC, Brazil) in the middle of the right ear and one in the left ear (between the upper and lower cartilage ridges), for the later placement of one antiparasitic and two identification ear tags, fitted only after complete healing of the wounds. A larvicidal and repellent product (Cidental^®^, Bimeda^®^, Monte Mor, SP, Brazil) was applied to the holes, navel dipping was repeated, and the calf was weighed on a calibrated scale (G Life, CA7000, São Paulo, SP, Brazil).

Calves in the WTS group received tactile stimulation, where the cowhand, who was restraining the calf, firmly rubbed both hands calmly and continuously over the lateral region of the body, front legs, and neck of the calf. Tactile stimulation was paused whenever necessary to ensure proper restraint.

### 2.3. Body Movements and Facial Expression Assessments

Five body movements and seven facial expressions categories (Table 1 and Table 2, respectively) were recorded on video (Sony^®^, Full HD Digital, CX405, Miyagi, Japan) using cameras strategically placed on tripods. All scoring was performed by a single trained observer through video analysis.

To assess intra-observer reliability, a weighted Kappa coefficient (κ) was calculated. The observer evaluated 20 videos randomly selected on two different days, one week apart. Most Kappa coefficients were above 0.61 (see Appendix A Table A1), which is interpreted as very good when between 0.81 and 1.0 or good (0.61–0.80) [30].

Since facial expressions were recorded with the camera focused on the calf’s face, the observer was blinded to the treatment groups (WTS and NTS). However, body movements recordings included full-body view, making it possible to see when tactile stimulation was being applied.

**Table 2:** Descriptions of the seven facial expression categories considered in the study (adapted from Gleerup et al. [31]).

Facial Expression Categories	Scores	Descriptions
Sclera	0	Sclera nor visible
	1	Sclera visible at least once
Tension above the eye area	0	No expression lines above the eye area
	1	Expression lines above the eye once
	2	Expression lines above the eye more than once
Eye-opening	0	Eye open without eyelid contraction
	1	Eye closed with eyelid contraction, blinks once
	2	Eye closed with eyelid contraction, blinks more than once
Third eyelid	0	Not visible
	1	Visible once
	2	Visible more than once
Tension of facial muscles	0	No expression lines on the face
	1	Expression lines on the face once
	2	Expression lines on face more than once
Strained nostrils	0	No expression lines around nostrils
	1	Expression lines around nostrils once
	2	Expression lines around nostrils more than once
Mouth opening	0	Mouth closed
	1	Mouth opened once
	2	Mouth opened more than once
	3	Mouth opened with the tongue out once
	4	Mouth opened with the tongue out more than once

### 2.4. Qualitative Behavior Assessment

The qualitative behavior assessment was (QBA) conducted as described by Wemelsfelder et al. [32], aiming to identify the emotional state of the calves. The assessment included 18 terms (Relaxed, Fearful, Agitated, Calm, Content, Indifferent, Frustrated, Bored, Tense, Inquisitive, Irritable, Uneasy, Apathetic, Happy, Distressed, Lively, Pleased, and Comfortable), adapted from Welfare Quality^®^ [33] and Ceballos et al. [34]. These terms were measured using a 125 mm visual analog scale, where the minimum value indicates the absence of the specific emotional state, and the maximum value represents its highest expression.

A previously trained observer with high intra-observer reliability assigned the ratings by watching video recordings, focusing on the face of each calf, and recording for 30 s after the completion of the handling procedures.

Intra-observer reliability was assessed by calculating the Intraclass Coefficients of Correlation (ICC). For most terms (Relaxed, Agitated, Calm, Content, Tense, Inquisitive, Irritable, Uneasy, Happy, Lively, and Pleased), the ICC was very good. For Fearful, Frustrated, Apathetic, Distressed, and Comfortable, it was classified as good [30]. The terms Indifferent and Bored were removed from the data analysis due to an ICC below 0.61 (see Appendix A Table A2).

### 2.5. Heart Rate Assessment

Heart rates (HRs) were measured using a heart rate sensor (Polar H9, Polar Electro, Kempele, Finland) positioned on a chest strap fixed around each calf’s chest after separating it from its mother until the end of the second tactile stimulation. Conductive gel (Spin Power™ Heart Rate Monitor Conductive Gel, 5-Ounce, Greensboro, NC, USA) was applied to improve contact. The sensor was activated through a mobile application (Polar Beat, Polar Electro, Kempele, Finland) recording heart rates every 0.3 s.

Mean heart rates were estimated considering three moments: HR1 = while the calf was restrained and positioned in lateral recumbency on a cushion; HR2 = during identification procedures; and HR3 = after completion of identification procedures. The differences between HR3 and both HR1 and HR2 were calculated.

### 2.6. Performance Assessment

The calves were weighed after finishing handling procedures (initial weight) and again at weaning (~8 months of age). Based on these data, average daily weight gain (ADG) and weaning weight adjusted to 240 days (WW240) were calculated using the formula:

$$WW240=\frac{WW-IW}{AW}\times 240+IW$$

where WW = weaning weight, IW = initial weight, and AW = age at weaning (in days).

### 2.7. Statistical Analyses

The categories “mouth opening”, “vocalization”, and “sclera” were excluded from the analyses due to low variability (more than 90% of the animals did not exhibit these behaviors). Some scores of two body movements (“attempt to escape”, and “breathing”) and four facial expression categories (“eye opening”, “tension above the eye area”, “third eyelid”, “tension of facial muscle”) were observed at very low frequencies (less than five occurrences). Therefore, their scores were reorganized as follows: “attempt to escape”, 0 = no attempt to escape and 1 + 2 = one or more attempt to escape; “breathing”, 0 = rhythmic breathing no audible and 1 + 2 + 3 + 4 + 5 = audible rhythmic or arrhythmic breathing at least once; “eye opening”, 0 = eye open without eyelid contraction and 1 + 2 = eye closed with eyelid contraction at least once; “tension above eye area”, 0 = eye open without eyelid contraction and 1 + 2 = eye closed with eyelid contraction at least once; “third eyelid”, 0 = no visible and 1 + 2 = visible at least once; “tension of facial muscle”, 0 = no expression lines on the face and 2 = expression lines on the face more than once; and “strained nostril”, 0 = no expression lines around the nostrils and 2 = expression lines around the nostrils more than once.

Statistical analyses were performed in R software with an RStudio integrated development environment (R version 4.1.3., RStudio Inc., Northern Avenue Boston, MA, USA). Statistical significance was set at *p* < 0.05. 

To assess associations between body movements and facial expression categories with the treatments (WTS and NTS), chi-square (χ^2^) and Fisher exact tests were applied in a contingency table.

QBA data were subjected to a principal component analysis (PCA), a multivariate technique that reduces data dimensionality while retaining as much variance as possible [35]. Data were transformed into a new coordinate system, the principal components, which capture the largest variation in the data. Horn’s parallel analysis was used to determine the number of components to retain selecting those with adjusted eigenvalues > 1.0 [36]. Component loadings above 0.6 in absolute value were considered the most relevant [33]. The third component, despite having an eigenvalue above 1.0, was excluded as all loadings were below <0.4 [37]. Based on the results of this analysis, we calculated the indexes obtained by each calf in principal component 1 (PC1) and 2 (PC2).

Generalized linear models were applied to evaluate the fixed effects of treatment (WTS and NTS), sex (female and male), and their interactions on heart rate measurements (HR1, HR2, HR3, HR3-HR1, and HR3-HR2), PC1 and PC2 indexes, ADG, and WW240. For ADG and WW240. Initial body weight was included as covariate for ADG and WW240. One female from the NTS group was excluded from ADG and WW240 analyses due to missing weaning data.

Model selection was performed using the Akaike Information Criterion (AIC) and the Bayesian Information Criterion (BIC). Residual errors were assessed for normality using a quantile–quantile plot, histogram, and the Shapiro–Wilk test. The HR3-HR2 difference required a square root transformation but is presented in the original scale.

Multiple comparisons were conducted using Tukey’s test for adjusted means. Outliers were identified and retained as they represented individual variability. Box plots were created to illustrate comparisons in the original variable scale.

Pearson correlation coefficients were estimated to assess relationships between heart rate variables (HR1, HR2, HR3, HR3-HR1, and HR3-HR2), PC1 and PC2 indexes, and performance variables (ADG and WW240).

## 3. Results

### 3.1. Body Movements and Facial Expression Assessments

There were significant (*p* < 0.001) treatment effects on only one type of body movement (“head movements”) and in three facial expressions categories (“third eyelid”, “eye-opening”, and “strained nostrils”). For most of these traits—except for “strained nostrils”—a higher percentage of WTS calves had higher scores (Table 3).

### 3.2. Qualitative Behavior Assessment (QBA)

**Figure 1:**
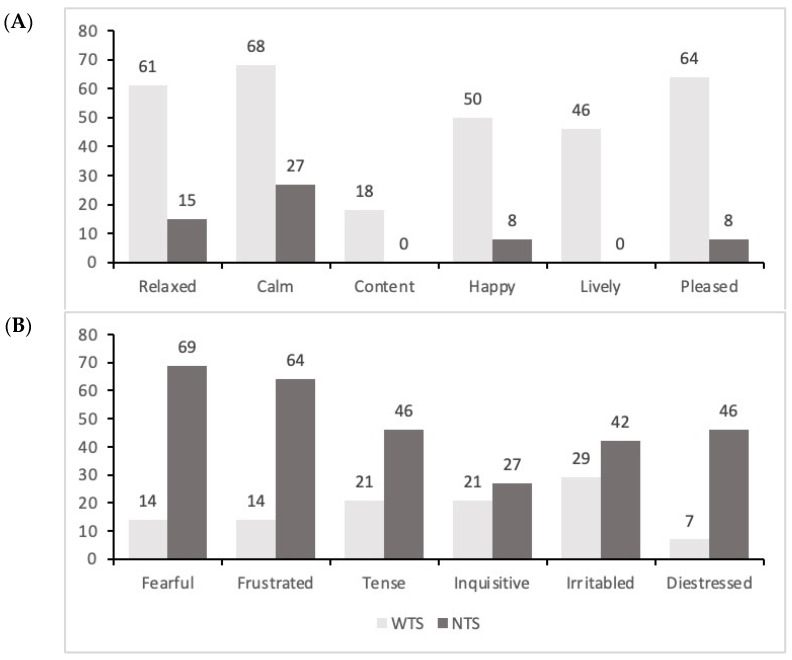
Percentages of calves showing PC1 terms rated above 51 mm on the visual analog scale, categorized as either positive ((**A**): Relaxed, Calm, Content, Happy, Lively, and Pleased) or negative ((**B**): Fearful, Frustrated, Tense, Inquisitive, Irritable, Distressed) valences, according to the treatments.

The two principal components (PC1 and PC2) explained 82.89% of the total variance. PC1 (*x*-axis) explained 63.01%, with high negative loading for the terms Fearful, Frustrated, Tense, Inquisitive, Irritable, and Distressed and positive for Relaxed, Calm, Content, Happy, Lively, and Pleased (Table 4, Figure 1). PC2 (*y*-axis) explained 19.88% of the total variation, with high negative loadings for Agitated and Uneasy and positive loadings for Apathetic and Comfortable (Table 4).

Based on these results, we labeled PC1 “emotionality” as it reflects the positive and negative emotional states of calves. In turn, PC2 was labeled “excitability” as it represents the general level of activity, ranging from the most apathetic animals to the more agitated and uneasy ones.

It was observed that a higher and a lower proportion, respectively, of WTS calves were rated with scores above 51 mm on the visual analog scale in terms of positive (Relaxed, Calm, Content, Happy, Lively, and Pleased) and negative (Fearful, Frustrated, Tense, Inquisitive, Irritable, and Distressed) valences, respectively (Figure 1).

**Figure 2:**
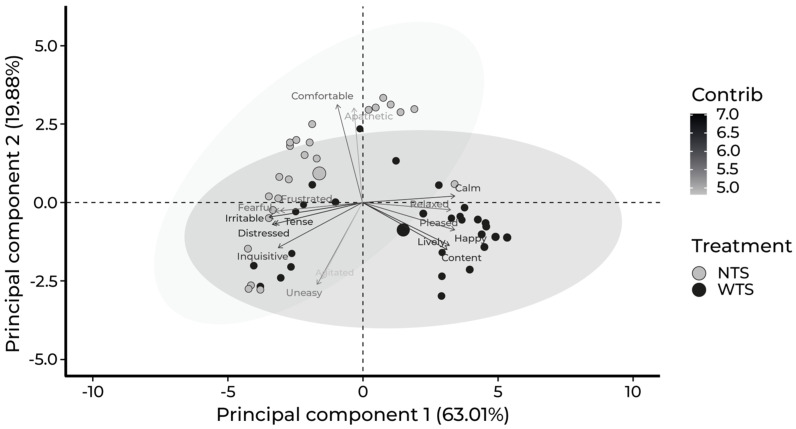
Plots of the loadings for the 16 terms of the qualitative behavior assessment (QBA) and the distribution of calves in each quadrant of the principal component analysis (PC1 and PC2) according to the treatments. WTS = tactile stimulation (black circles) and NTS = no tactile stimulation (gray circles).

The distribution of animals in the plane according to the two principal components (PC1 and PC2) and broken down by treatment is shown in Figure 2. Gray circles represent the NTS calves, while black circles represent WTS ones. Notably, most WTS calves are positioned mainly in quadrant 2 (indicating positive emotional state and high excitability) with some distributed across other quadrants. In contrast, most NTS calves are mainly positioned in quadrant 4 (indicating negative emotional state and low excitability), with a smaller number appearing in quadrants 1 and 3.

A significant effect of the treatment was found on PC1 and PC2 indexes, but not on sex and the interaction between the treatment and sex. The means and respective standard deviations of PC1 and PC2 categorized by treatment are presented in Table 5.

### 3.3. Heart Rate Assessment

There was no significant effect (*p* > 0.05) of treatment, sex, and treatment–sex interaction on heart rate assessed in the first and second periods (Table 5). However, there was a significant difference (*p* < 0.05) between treatments and sex for HR3-HR2, with the WTS calves showing a lower mean than the NTS calves and males a lower mean than females (Table 5). HR3 and HR3-HR1 showed a trend (*p* = 0.06), with WTS calves having lower HR3 and HR3-HR1 values than NTS calves (Table 6).

Additionally, in the final period of tactile stimulation, 77% of the calves in the WTS group showed a reduction in heart rate, compared to only 24% of the NTS calves.

### 3.4. Performance Assessment

**Figure 3:**
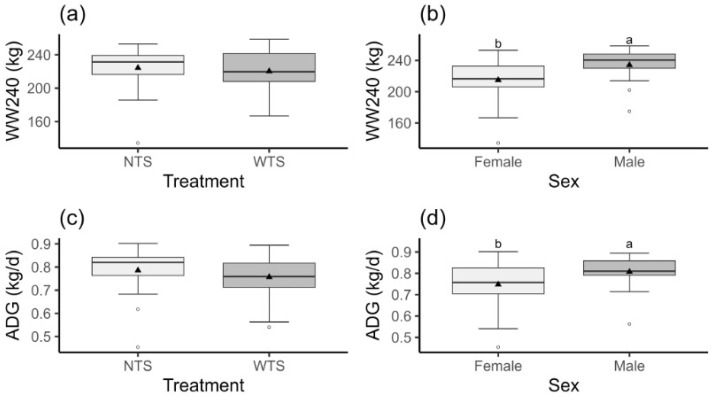
Box plots of weaning weight adjusted to 240 days of age (WW240, kg) and average daily gain (ADG, kg/d) of Nellore calves according to the treatment ((**a**,**c**): WTS = tactile stimulation and NTS = no tactile stimulation) and sex of the calves ((**b**,**d**): female and male). Different letters indicate significant differences between treatments (*p* < 0.05). Triangles represent means and circles outliers.

There was no significant effect (*p* > 0.05) of treatment and treatment–sex interaction on ADG and WW240. However, significant differences in the performance traits were observed between the sexes, with males showing higher ADG and WW240 than females (Figure 3).

No significant correlation was found between the performance indicators (WW240 and ADG) and the PC1 and PC2 indexes, nor between these indexes and HR1, HR2, HR3, and HR3-HR1. However, a significant positive correlation was observed between the PC2 index and HR3-HR2 (r = 0.53, *p* < 0.05), indicating that calves with a higher negative PC2 index (predominantly WTS calves, see Figure 1) tend to show a greater reduction in heart rates during the final period of tactile stimulation.

## 4. Discussion

The results indicate that tactile stimulation has the potential to improve calves’ welfare in the short-term. Most WTS calves were classified (via the QBA) as having positive emotionality (Relaxed, Calm, Content, Happy, Satisfied, and Pleased) when compared to NTS calves, which exhibited more negative valences (Fearful, Frustrated, Tense, Inquisitive, Irritable, and Distressed). Additionally, despite the short time interval between HR measurements, WTS calves showed a greater reduction in HR than NTS calves. The lack of differences in ADG and WW240 between WTS and NTS calves suggests that short-term tactile stimulation in a calf’s early life has only a temporary positive effect on welfare. These results support Adcock and Tucker’s [26] statement that tactile stimulation applied during routine handling procedures is positive reinforcement manifesting in the expression of positive emotional states.

The results regarding body movement and facial expression indicators were inconclusive. For example, vocalization is often reported as a pain-related expression [31,38,39], but it was not observed in most calves assessed in this study. Thus, one could mistakenly conclude that the low frequency of vocalization in this study indicates that the handling procedures did not cause pain, which was likely not the case. Additionally, the “tension of facial muscle” and “tension above the eye area”, which are facial expression indicators commonly used to assess pain [31,40], were observed in fewer than 50% of the calves and did not differ between treatments. However, it should be noted that Grant [41] found a low frequency of these facial expressions during ear tattooing compared to castration and tail docking in lambs. Thus, the facial expression categories used in this study as pain indicators may not be the most suitable for assessing the welfare of calves subjected to first handling procedures, regardless of tactile stimulation.

Regarding the “sclera” facial expression category, which was present in most calves, it can be interpreted as an indicator of the calves’ reactions in relation to the cowhand, as reported by Grandin and Deesing [42], who described that herd animals orient their eyes and ears toward the object, person, or some new sound. Since the handling procedures were carried out with the calves kept in lateral recumbency, they most likely attempted to look in the direction of the cowhand positioned behind their heads, making the “sclera” visible. Another possible explanation for the high percentage of animals with apparent “sclera” is a heightened stress response (excitability), as reported by Sandem et al. [43] and Core et al. [44]. However, it should also be considered that part of the “sclera” is naturally visible most of the time. A greater proportion is expected to be visible in response to aversive stimuli, while a smaller proportion may be observed when in the presence of a positive stimuli [43]. The calves’ attempts to keep the cowhand within their visual field may also explain the “head movements”, which were common among the animals. Furthermore, “head movements” and “third eyelid”—generally characterized as a searching expression [42]—were observed more frequently in WTS calves. This led us to interpret these behaviors as attempts by the calves to keep the cowhand in their visual field. Similar results were reported by Schmidek et al. [4] in horses, describing that 60% of the foals that accepted human approach exhibited similar reactions. However, these findings (and the probable explanation for their higher frequency in WTS calves) do not align with the “eye-opening” observations, as WTS calves exhibited closed eyes with eyelid contraction more frequently than the NTS calves. On the other hand, “strained nostrils”—one of the tension and pain indicators [31]—occurred more frequently in NTS calves, leading us to hypothesize that tactile stimulation may induce relaxation in calves.

Previous studies [45,46,47] have shown that the QBA is an appropriate tool for assessing positive and negative emotional states in animals and identifying individual differences in their responses. Our results support these findings, as we observed individual variation differences in how calves reacted to handling procedures, including tactile stimulation. This variation fits well with the concept of the dimensional theory of emotions, as proposed by Russell [48], which describes the circumplex model based on subjective feelings. This model allows us to classify our calves according to their emotionality (pleasant or unpleasant) or excitability (activation or deactivation).

The high proportion of WTS calves exhibiting a positive emotional state and high excitability may have been triggered by the receipt of a reward (e.g., tactile stimulation) or a motivational state [49,50], characterized by signals indicating that the body is returning to equilibrium [49]. Alternatively, these responses could be attributed to a “searching state”, an emotion linked to the activation of one of the brain’s basic emotional circuits, present from the first day of life and critical for survival [51].

Although individual differences in calf reactions can be attributed to temperament [52,53] or coping style [54,55], the limited prior experience of these calves in interacting with humans—only occurring during first handling on the day of birth—prevents us from drawing definitive conclusions. Since the behavioral assessment was conducted only once, it is impossible to determine whether these reactions persist over time, a requirement for classifying temperament traits. Other possible explanations for individual differences in behavior include the quality of cowhand–calf interactions, which can influence behavioral, physiological, and neuroendocrine responses to handling procedures. To attribute the high excitability of the animals solely to temperament would require a different study design. However, as reported by Destrez et al. [56], not all calves react positively to gentle handling.

Brscic et al. [46] obtained similar results when assessing the associations between the QBA and clinical indicators in beef calves. They found that terms associated with positive emotions (Relaxed, Friendly, Sociable, and Happy) and negative emotions (Fearful, Agitated, Tense, Frustrated, Restless, Apathetic, and Distressed) had high but opposing signs on PC1, whereas terms related to excitability (Active, Playful, Excited, Inquisitive, and Aggressive) and boredom (Depressed, Indifferent, and Bored) had high positive and negative loadings, respectively, on PC2. Positive terms on PC2 indicate indifference, while negative terms indicate excitement.

Heart rates were high in all calves, likely due to their young age and the stress caused by the handling procedures to which they were exposed. Newborn mammals have a low heart volume, requiring the heart to pump at higher rates to maintain cardiac output [57,58]. Additionally, stress responses were likely induced by separating the calves from their mothers, restraining them, and carrying out the handling procedures [59]. Thus, it is reasonable to assume that stress induced by handling results in a greater release of catecholamines, consequently increasing heart rate [60,61].

Although there was no statistical effect of treatment on HR1, HR2, and HR3, the WTS calves had lower heart rate means than the NTS calves. These results, combined with the greater reduction in heart rates observed after handling procedures in WTS calves (HR3-HR1 and HR3-HR2), may be explained by the relaxation response observed in cattle when licked, which has been associated with a calming effect [62,63,64]. This suggests that tactile stimulation may have reduced stress by inducing acute antinociceptive effects likely associated with oxytocin release, which influences pain control systems [65,66].

It is expected that tactile stimulation in early life positively impacts growth [10,19]. However, this was not the case in our study, as we did not find a significant effect of tactile stimulation on performance traits. This aligns with findings in beef calves that received tactile stimulation only once at the beginning of their lives [67] and in lambs [68] that were stimulated three or five times a week from birth to weaning. In both cases, however, the authors found that the stimulated animals exhibited behavioral changes that favored human–animal interaction.

A similar result was reported by Pontes et al. [69] in dairy cattle subjected to handling practices (including tactile stimulation) from birth to weaning. The authors reported enduring benefits, such as reducing fear responses toward humans, but no significant impact was reported on the heifers’ health or growth. Furthermore, the authors observed that the positive behavioral effects diminished over time.

Tactile stimulation applied to neonates has been shown to promote bonding between parents and offsprings [70]. So, why would it not also foster a bond between humans and calves? If that was the case, it could potentially influence the development of long-term relationships and, consequently, calf performance. However, our results indicate that a very short, single application of tactile stimulation early in a calf’s life during handling procedures is insufficient to induce a human–calf bond and, therefore, does not significantly affect long-term calf welfare.

## 5. Conclusions

Tactile stimulation of newborn Nellore calves has the potential to promote their short-term welfare to a limited extent, but without impairing the efficiency of standard handling procedures. However, the individual differences in how calves respond to tactile stimulation should be further explored in future studies. Additional research is also needed to assess the quality of human–animal interactions during handling procedures, including tactile stimulation, and to check its short- and long-term effects on cattle welfare.

## Figures and Tables

**Table 1:** Descriptions of the five body movements categories considered in the study.

Body Movements Categories	Scores	Descriptions
Head movements	0	Stands still all the time
	1	Pushing sideways once
	2	Pushing sideways more than once
Attempts to escape	0	No attempt to escape
	1	One attempt to escape
	2	More than one attempt to escape
Breathing	0	Rhythmic breathing, no audible
	1	Rhythmic breathing, easily audible once
	2	Rhythmic breathing, easily audible more than once
	3	Arrhythmic breathing, easily audible once
	4	Arrhythmic breathing, easily audible more than once
	5	Arrhythmic breathing all the time
Vocalization	0	No vocalization
	1	Vocalize at least once
Swallowing	0	No swallowing
	1	Swallowing at least once

**Table 3:** Relative frequencies (%) of body movements and facial expression categories showing associations with treatments (where WTS = tactile stimulation (n = 28) and NTS = no tactile stimulation (n = 26)), analyzed using chi-square * or Fisher’s exact tests **.

Body Movements Categories	WTS (%)	NTS (%)	*p*-Values
Head movements *			<0.001
Stand still all the time	46	61	
Pushing sideways at least once	18	31	
Pushing sideways more than once	36	8	
Facial expression categories			
Third eyelid **			<0.001
No visible	25	73	
Visible at least once	75	27	
Eye-opening *			
Eye open without eyelid contraction	21	50	<0.001
Eye closed with eyelid contraction at least once	18	31	
Eye closed with eyelid contraction more than once	61	19	
Strained nostrils **			<0.001
No expression lines around nostrils	43	12	
Expression lines around nostrils at least once	57	88	

**Table 4:** Adjusted eigenvalues and variances and loadings obtained in the principal component analysis for each one the 16 terms used in the qualitative behavior assessment. Loadings in bold represent the higher values (above 0.6 in absolute value) of each principal component (PC1 and PC2).

Item	PC 1 (Emotionality)	PC 2 (Excitability)
**Adjusted eigenvalues**	**9.00**	**2.36**
**Variances**	**63.01%**	**19.88%**
**Loadings**		
Relaxed	**0.89**	−0.06
Fearful	**−0.90**	−0.08
Agitated	−0.42	**−0.68**
Calm	**0.93**	0.06
Content	**0.86**	−0.41
Frustrated	**−0.84**	−0.07
Tense	**−0.90**	−0.19
Inquisitive	**−0.86**	−0.40
Irritable	**−0.95**	−0.13
Uneasy	−0.46	**−0.71**
Apathetic	−0.09	**0.83**
Happy	**0.88**	−0.38
Distressed	**−0.92**	−0.19
Lively	**0.86**	−0.41
Pleased	**0.94**	−0.24
Comfortable	−0.26	**0.86**

**Table 5:** Adjusted means and respective standard deviations of PC1 and PC2 indexes according to treatments (WTS = tactile stimulation and NTS = no tactile stimulation) and sex (female and male) in Nellore calves. Means followed by different letters differ significantly (*p* < 0.05).

Treatment		Sex	
WTS	NTS	Male	Female
**PC1 index**			
1.58 ± 0.55 ^a^	−1.69 ± 0.58 ^b^	0.01 ± 0.63	−0.12 ± 0.48
**PC2 index**			
−0.94 ± 0.29 ^b^	0.77 ± 0.31 ^a^	−0.51 ± 0.34	0.35 ± 0.26

**Table 6:** Means and respective standard errors of heart rates (HR, bpm) recorded at three time points (HR1 = when a calf was being restrained and positioned in lateral decubitus on a cushion, HR2 = during identification procedures, and HR3 = after completion of identification procedures) according to the treatments and sex of the Nellore calves. Different letters indicate statistical differences between treatments or sex (*p* < 0.05) and * indicates *p* = 0.06. WTS = tactile stimulation and NTS = no tactile stimulation.

HR (bpm)	Treatment		Sex	
	WTS	NTS	Female	Male
HR1	173 ± 5.40	180 ± 5.44	172 ± 4.78	180 ± 6.00
HR2	166 ± 4.71	171 ± 4.96	162 ± 4.19	174 ± 5.41
HR3	159 ± 4.60 *	172 ± 4.84	164 ± 4.09	167 ± 5.27
HR3-HR1	−14.49 ± 3.26 *	−6.34 ± 3.28	−8.73 ± 2.88	−12.10 ± 3.62
HR3-HR2	−6.28 ± 2.58 ^b^	1.46 ± 2.72 ^a^	1.31 ± 2.36 ^a^	−6.20 ± 2.99 ^b^

## Data Availability

Restrictions apply to the availability of these data. Data will be available on request from the corresponding author after the permission of the Orvalho das Flores farm owner.

## Appendix A

**Table A1:** Results from the intra-observer reliability test for body and facial expression variables.

Body and Facial Expressions Categories	Kappa Reliability Test
**Body expression**	
Head position	0.90
Breathing	0.94
Reaction to escape	1.00
Vocalization	1.00
Swallowing	1.00
**Facial expressions**	
Orbital tightening	0.61
Eye white showing	1.00
Tension above eye	0.75
Eye tightness	0.87
Third eyelid	0.63
Tension masticatory muscle	0.97
Opening mouth	1.00

**Table A2:** Results from the intra-observer reliability test for the qualitative behavior assessment (QBA) data.

QBA Terms	Intraclass Coefficients of Agreement
Relaxed	0.90
Fearful	0.78
Agitated	0.88
Calm	0.86
Content	0.82
Frustrated	0.77
Tense	0.84
Inquisitive	0.90
Irritable	0.88
Inquisitive	0.82
Apathetic	0.62
Happy	0.84
Afflicted	0.79
Animated	0.81
Satisfied	0.90
Comfortable	0.71
